# Training Community–Academic Teams to Use Low-Cost Air Monitors: A Mixed Method Evaluation of the RISE Communities Program

**DOI:** 10.3390/ijerph23081051

**Published:** 2026-08-13

**Authors:** Mackenzie Martin, Daniel Hargraves, Patrick Ryan, Jacqueline Knapke

**Affiliations:** 1 College of Medicine, University of Cincinnati, Cincinnati, OH 45267, USA; 2 Department of Family and Community Medicine, University of Cincinnati, Cincinnati, OH 45267, USA; hargradm@ucmail.uc.edu (D.H.); knapkeje@ucmail.uc.edu (J.K.); 3 Division of Biostatistics and Epidemiology, Cincinnati Children’s Hospital Medical Center, Cincinnati, OH 45267, USA; patrick.ryan@cchmc.org; 4 Department of Pediatrics, Division of Biostatistics and Epidemiology, University of Cincinnati, Cincinnati, OH 45267, USA

**Keywords:** environmental health research training, community-based participatory research, air quality

## Abstract

**Highlights:**

**Public health relevance—How does this work relate to a public health issue?**

- This study shows one way to increase the use of low-cost air sensors for community monitoring of air quality.
- Teaching citizens how to monitor their air quality and make decisions for their own health is important.

**Public health significance—Why is this work of significance to public health?**

- A short training program with low-cost monitors can be a simple and cost-effective solution for future community air monitoring programs.
- This study shows an example of the effectiveness of teaching the community how to engage in air monitoring, empowering communities to take action.

**Public health implications—What are the key implications or messages for practitioners, policy makers and/or researchers in public health?**

- Community-engaged participatory research should be a priority for public health efforts because it helps increase awareness and participation in public health issues.
- Future programs that train community members and academics on air monitoring should know that data handling continues to be a barrier for many individuals.

**Abstract:**

Concerns regarding poor air quality in communities experiencing disproportionately high levels of air pollution frequently motivate formation of community–academic partnerships. Low-cost air monitors can assess air pollution but require specific training. The RISE Communities program was created to support partnerships in air quality training and community-engaged research. The RISE Communities program in-person training took place in Cincinnati, OH, in summers 2023 and 2024. The training included hands-on workshops, lectures, and monthly webinars with continued expert guidance for one year following the in-person training. A mixed method evaluation included pre-, post-, and one-year follow-up surveys, webinar evaluation surveys, and focus groups at the conclusion of each cohort. Participants reported significantly increased confidence in describing the health impacts of air pollution and using low-cost air sensors. Subjective feedback commented on the need for more tailored and interactive webinars and data training. Participants agreed that the goals of the RISE Communities training program were met and skills were sustained. This mixed method evaluation study demonstrates that an immersive training program for academic and community partners resulted in significantly higher confidence levels related to community partnership, data analytics, data visualization, and achieving project planning outcomes.

## 1. Introduction

The significant negative impacts that air pollution has on human and environmental health have been extensively described and include cardiopulmonary, neurologic, skin, and endocrine disease [1,2,3,4,5]. Globally, exposure to air pollution, including fine particulate matter (PM_2.5_), is responsible for more than 7.5 million deaths, including nearly 70,000 deaths per year in the United States (US) [6]. Recently, the US Environmental Protection Agency (US EPA) adopted a lower annual average air quality standard for PM_2.5_ (9 µg/m^3^); compliance with this limit and other National Ambient Air Quality Standards (NAAQS) is determined through a network of regulatory monitoring stations. A recent analysis of NAAQS monitoring locations estimates that more than 40% of urban locations, representing approximately 20 million residents, will exceed the new standard but be undetected due to the sparsely distributed locations of the current monitoring sites [7]. Notably, inadequate monitoring disproportionately impacts people of color and lower-income individuals [8,9].

In the past decade, low-cost air monitors have been deployed by community residents and researchers to address the limitations of regulatory monitoring networks. These monitors are increasingly accurate, reliable, and economical, and their deployment in impacted communities may help residents identify specific sources of poor air quality and motivate policy changes based on improved awareness [10,11,12,13,14,15]. While low-cost sensor networks are being established in some communities, widespread application of this technology remains limited due to challenges in sensor selection, maintenance, calibration, data collection and analysis, interpretation, and other technical barriers faced by users [13,16,17,18]. Additionally, limitations of low-cost air monitors vary by device and may include decreased accuracy and precision compared to regulatory monitors, but these may be addressed by colocation, calibration, and other methods of data correction [19].

Community-based research projects across the US have shown much promise in integrating the technical science of air quality research with the advocacy and policy needs of the communities involved in these projects [20]. Current projects have evaluated the interactions between researchers and policy makers as well as teaching young people the basics of air quality monitoring [21,22]. Often, participants in these projects indicate the need for additional technical training and time to establish trust between academic and community partners. There are few formal training programs that develop these technical and teamwork skills for community-engaged research partnerships. Furthermore, evaluation of the effectiveness of a formal training program has not been discussed in the literature before.

Our Research Innovations using Sensor Technology to Improve Community Health (RISE Communities) training program is motivated by this need and serves as one of the first programs to offer formal training in air quality monitor installation, data extrapolation, data visualization, team building, and best practices for community engagement. The program was intentionally designed for teams of community members and academic partners and includes formal evaluation of participant feedback throughout the training program to guide program improvement and recommendations for future, similar training programs. Our program goals include: (1) foster community–academic partnerships through research education, training, and team development activities, (2) provide technical training in the application of low-cost sensors for indoor, outdoor, and personal air monitoring in communities, and (3) establish a community of practice to address air quality in communities nationwide. The objective of this report is to describe the RISE Communities training program and evaluate its effectiveness to increase confidence and skills in community engagement, team function, and air sensor data collection and analysis in the program participants.

## 2. Materials and Methods

### 2.1. Participant Recruitment

Participants applied to join the RISE Communities program, and eligible applications included a research team comprised of at least one member from an academic institution and one member associated with a community organization or living in a community impacted by poor air quality. Teams were not formed by the RISE Communities program, but applicants were recruited through advertisements and dissemination of program information on the RISE Communities website (https://www.ejsensors.com/ accessed on 9 August 2026), professional networks including the National Institute of Environmental Health Sciences (NIEHS) Partnerships for Environmental Public Health (PEPH) newsletter, and colleagues and collaborators at institutions and community organizations throughout the US. RISE Communities faculty members also distributed information about the program to their networks. The RISE Communities program directors presented an overview of the program at the annual PEPH meeting held at NIEHS, the Association of Clinical and Translational Science (https://www.actscience.org/ accessed on 9 August 2026) meeting held in Las Vegas, NV, and the Air Sensors International Conference (https://asic.aqrc.ucdavis.edu/ accessed on 9 August 2026) held in Riverside, CA, all held in 2024. Team applications included a project proposal where the applicants described their project rationale, community setting and concerns, goals, project plan, and timeline. Applications were received online through the RISE Communities website and reviewed and scored by the program directors and program manager based on the team’s qualifications, needs, and alignment with RISE Communities program goals.

### 2.2. In-Person Training

**Figure 1:**
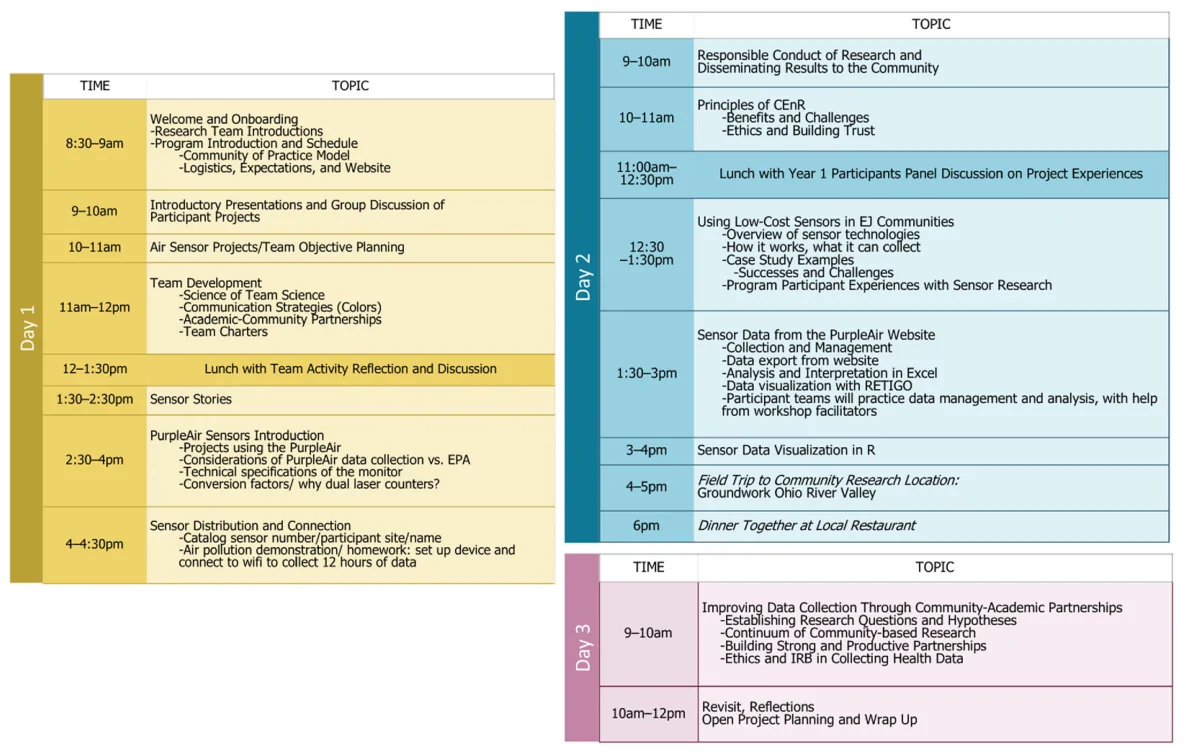
A layout of the schedule for the RISE Communities in-person training program for cohort 2. The types of topics and activities can be seen in the daily itinerary within this schedule layout.

In-person training sessions were held in Cincinnati, OH, during the summers of 2023 (cohort 1) and 2024 (cohort 2). Each session consisted of a two-and-a-half-day program led by faculty, staff, professionals in the training topics, and sensor developers who are national experts in team science, community-engaged research, environmental health, epidemiology, and low-cost air monitors. Topics included principles of air pollution and health, choosing the right air sensor for your project, team development, principles of community-engaged research, engaging with communities using air sensors, sensor operation, data visualization, and responsible conduct of research. Local community partners provided a tour of a neighborhood in Cincinnati, OH, that is impacted by poor air quality and where a network of low-cost (PurpleAir, Bluffdale, UT) sensors was established to learn how they implement community learning and research in their work. Figure 1 provides the schedule for the in-person training components for cohort 2. All participants were also provided with ten PurpleAir sensors for the creation and deployment of an air sensor network in their project communities. The RISE Communities program did not actively engage in each team’s project, but rather provided the foundational knowledge, equipment, and additional training through webinars and consultations as needed once participants were deploying sensors (see Section 2.3). Participants completed walk-through demonstrations on setting up low-cost sensors, downloading data recorded by the sensors, and visualizing an analyzed version of these data. Dedicated time was provided for teams to describe their communities, projects, and goals for the use of low-cost air monitors and receive group feedback and suggestions from all cohort participants and faculty.

### 2.3. Webinars and Online Resources

**Figure 2:**
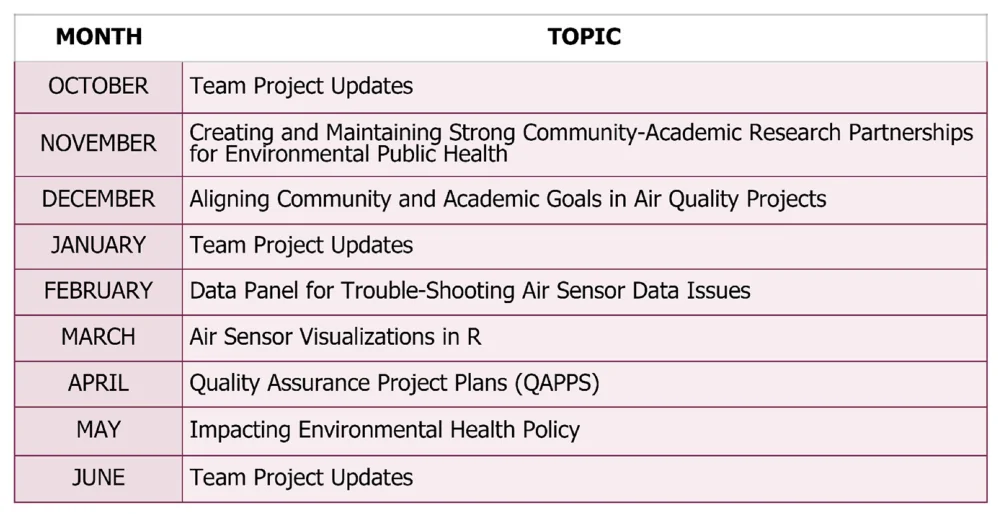
Schedule of RISE Communities webinars for cohort 2. A new topic was discussed each month and the topics are labeled next to their respective months.

After the in-person intensive education, monthly webinars were offered on follow-up topics that were suggested by trainees or that aligned with the status of their air sampling project implementation. All community–academic teams and faculty were invited to suggest topics and/or speakers for the webinar series based on interest and challenges faced by the participating teams. Webinar speakers included experts in data visualization, community-engaged research, public policy, and other topics. In addition, one webinar per quarter was reserved for teams to provide updates on their projects, receive peer feedback, and ask faculty and other attendees for suggestions or guidance. Figure 2 provides the webinar schedule for cohort 2 as an example of topics discussed in the months following the training. Webinars were recorded and posted to the program website. An online library of resources such as recordings of the in-person presentations, specific analysis methods and codes discussed during the training, a sensor calibration guide, and templates and examples of quality assurance project plans were also developed on the program website, as well as a discussion board to discuss common issues faced by teams and to share news stories. The participant teams were responsible for their own project-specific logistics, data collection, and analysis; results of their individual projects are outside the scope of this analysis.

### 2.4. Survey Data

Demographic information for the entirety of cohort 1 and cohort 2 was self-reported and collected through REDCap surveys when participants began the program.

We evaluated the effectiveness and outcomes of the RISE Communities training program using data collected in a pre-survey, post-survey, a survey collected one year after training was completed, webinar evaluation surveys, and focus group feedback collected from participants at the end of their training. Participants completed a pre-survey before attending the in-person training program in Cincinnati and a post-survey shortly after the training program concluded. Trainees were asked to rate their confidence on a 4-point scale correlating to the levels ‘Extremely confident (4)’, ‘Pretty confident (3)’, ‘A little confident (2)’, and ‘Not at all confident (1)’. The participants then completed the final survey approximately one year after participating in the in-person training program and once all training activities had concluded for their cohort. Table 1 provides a summary of the survey questions. Changes in confidence levels reported in these surveys were evaluated using paired *t*-tests, Friedman’s test (a non-parametric paired *t*-test), and effect sizes (Cohen’s *d*) to evaluate differences in the three related groups across different time points. The three time points include the pre-survey, the post-survey, and the end-of-year survey with 2 as degrees of freedom (*df*). Because only 22 participants completed the end-of-year survey, *n* = 22 for paired analyses purposes. Analyses were completed using Microsoft Excel (Redmond, WA, USA) and SPSS 29 (Armonk, NY, USA) software.

In addition to the confidence questions, the one-year survey inquired about how the training program contributed to their research outcomes, media representation, or community discussions. Additional questions, including open-ended items, were included to evaluate overall program effectiveness and sustainability of the research projects and partnerships, from the participants’ perspective. Table 2 provides a summary of additional questions included in the one-year follow-up survey. All survey data were collected through REDCap. The pre- and post-surveys were encoded to each individual participant along with demographic and contact information. However, the end-of-program survey was collected anonymously.

Trainees were also asked to complete anonymous evaluative surveys following each monthly webinar. Participants were able to slide a scale of 1–100 to indicate how strongly they agreed with statements about the quality of various aspects of the webinar, including value and usefulness, expertise of the presenter, engagement, and clarity of learning objectives.

### 2.5. Focus Groups

Focus groups were conducted by two different facilitators who were medical student research assistants and therefore unknown to participants, allowing for honesty and openness during the focus group discussion. They were conducted, recorded, and transcribed via Zoom approximately one year after the in-person training program had occurred, and shortly after the conclusion of the final webinar for each respective cohort. Participants were invited to share their perspectives on how RISE Communities impacted their individual projects and understanding of community-engaged environmental health research. The focus group guide is provided as Appendix A. Qualitative data obtained from the focus groups were analyzed using a thematic analysis approach where data were coded in order to construct thematic patterns across participant responses, with the intent of identifying key themes across cohorts. The medical student research assistants who conducted the focus groups were trained in qualitative analysis methods following the steps described by Creswell and Creswell, and they conducted initial analyses on the transcripts from cohorts 1 and 2 [23]. The transcripts and the initial results were then independently reviewed and refined by two RISE Communities program leads with extensive training and experience in qualitative methods. A negative case analysis was also performed to ensure credibility of the final findings. The qualitative dataset was small, so no software was used for analysis, but rather thematic results were organized and developed using a series of shared tables between the analysts following the iterative process described above.

## 3. Results

### 3.1. Participants

A total of 31 trainees participated in cohorts 1 and 2 of the RISE Communities program; however, only 22 participants completed the end-of-year survey. Of these, 58% were community partners, 32% academic partners, 7% local government officials, and 3% students at a university associated with the academic partner. The majority of participants (94%) reported having a bachelor’s degree or higher; 32% had a doctorate. Furthermore, participants were 74% women, 23% men, and 3% non-binary, transgender, or gender non-conforming. Participants reported their ethnicity as 13% Hispanic or Latino, and of those that reported their racial background, 29% reported being a minority. Two of the 31 participants reported having a disability by the ADA definition and three participants reported that English was not their first language. Finally, 45% of participants reported coming from a disadvantaged background. Table 3 provides a summary of participant demographics.

### 3.2. Survey Data

**Figure 3:**
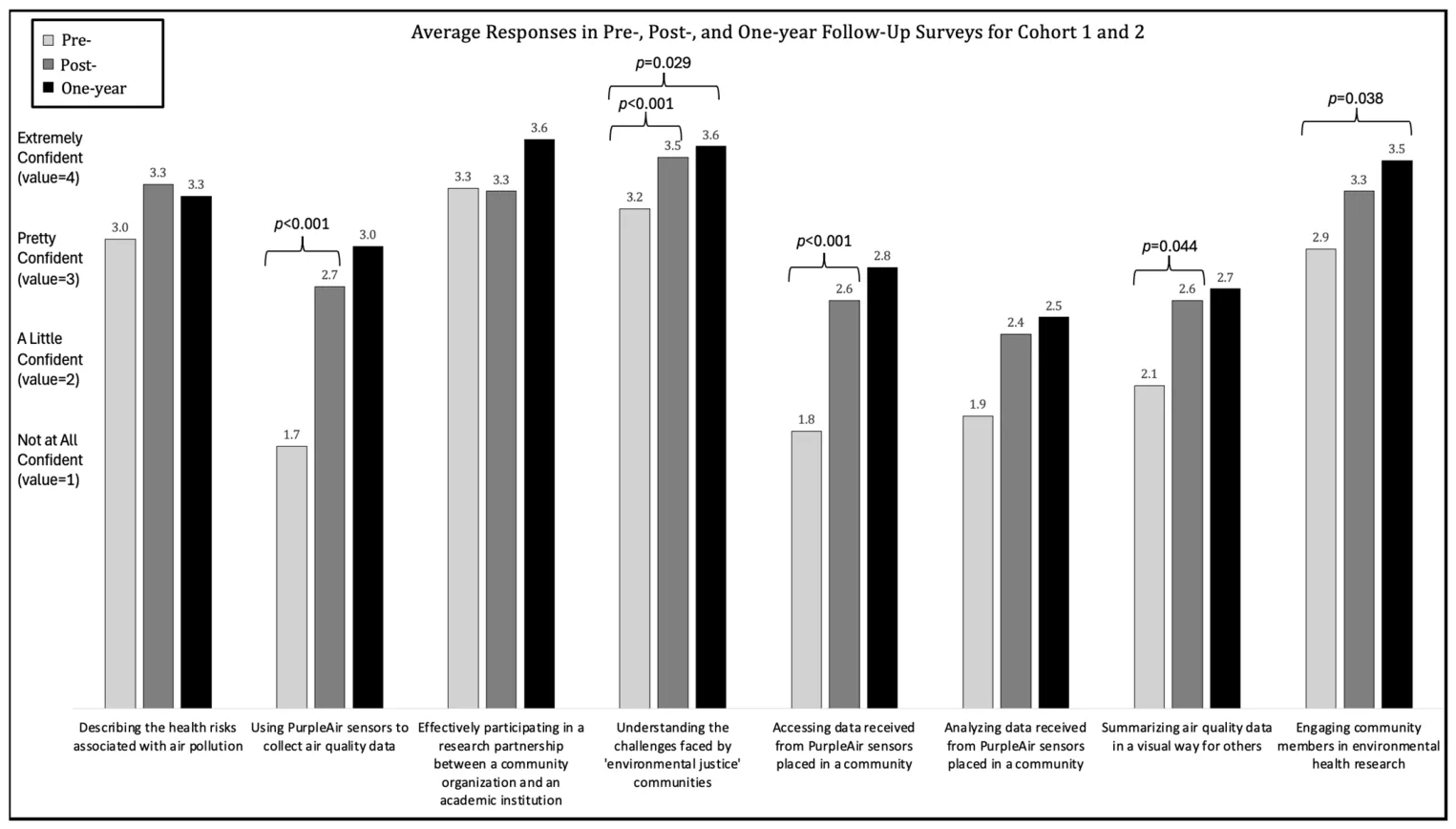
Confidence levels on pre-, post-, and one-year follow-up surveys among cohorts 1 and 2. Individual bars are labeled with the average numerical value reported by trainees for each statement in both cohort 1 and cohort 2. Trainees could rate their confidence in the statements found on the y-axis on a scale of 1 to 4. This scale is found on the x-axis. The pre-survey was completed before attending any training sessions. The post-survey was completed shortly after the training sessions were completed. The one-year survey was completed one year after the training was completed. The significance levels *(p* < 0.05) are labeled above the respective brackets.

The response rates of the pre- and post-surveys were 95% and 87%, respectively. The response rate for the one-year follow-up survey was 86%. Analyses of pre- and post-survey data show a significant increase in participants’ confidence levels across several survey domains (Figure 3). Results also show a significant increase in confidence levels between the pre-survey and the follow-up survey one year later. As shown in Figure 3, there was a significant increase (*p* < 0.05) in the confidence levels between pre- and post-survey responses for several statements: ‘Using PurpleAir sensors to collect air quality data’ (*p* < 0.001, *d* = 0.468, *df* = 2), ‘Understanding the challenges faced by ‘environmental justice’ communities’ (*p* < 0.001, *d* = 0.159, *df* = 2)), ‘Accessing data received from PurpleAir sensors placed in a community’ (*p* < 0.001, *d* = 0.335, *df* = 2), and ‘Summarizing air quality data in a visual way for others’ (*p* = 0.044, *d* = 0.162, *df* = 2)).

Participants reported continued significantly increased confidence (*p* < 0.05) in the one-year follow-up survey in several statements: ‘Understanding the challenges faced by ‘environmental justice’ communities’ (*p* = 0.029, *d* = 0.499, *df* = 2), and ‘Engaging community members in environmental research’ (*p* = 0.038, *d* = 0.203, *df* = 2). These confidence level responses are further outlined in Figure 3. A representative analysis of this data can be found in Supplementary Data Sheet S1.

Changes in confidence across trainee roles (community partners, local government officials, student participants, academic partners) are presented in Table 4. Overall, community members did not show significant increases in confidence levels in various statements that academic partners did show significantly increased confidence in. When the pre-survey is compared to the post-survey for only academic partners, confidence in the statement ‘Describing the health risks associated with air pollution’ increased by 0.67 points (95% CI [0.082, 1.25]), ‘Understanding the challenges faced by ‘environmental justice’ communities’ increased by 0.87 points (95% CI [0.23, 1.50]), and ‘Summarizing air quality data in a visual way for others’ increased by 1.03 points (95% CI [0.31, 1.75]) whereas community partners did not show similar trends. When the pre-survey is compared to the post-survey, both academic and community groups did show significant increases in confidence levels in the statements ‘Using PurpleAir sensors to collect air quality data’ and ‘Accessing data received from PurpleAir sensors placed in a community’, which are further outlined in Table 4. Furthermore, when the pre-survey is compared to the one-year follow-up survey, both groups also showed significantly increased confidence in ‘Using PurpleAir sensors to collect air quality data’, ‘Accessing data received from PurpleAir sensors placed in a community’, and ‘Analyzing data received from PurpleAir sensors placed in a community’. These confidence levels are further described in Table 4.

**Figure 4:**
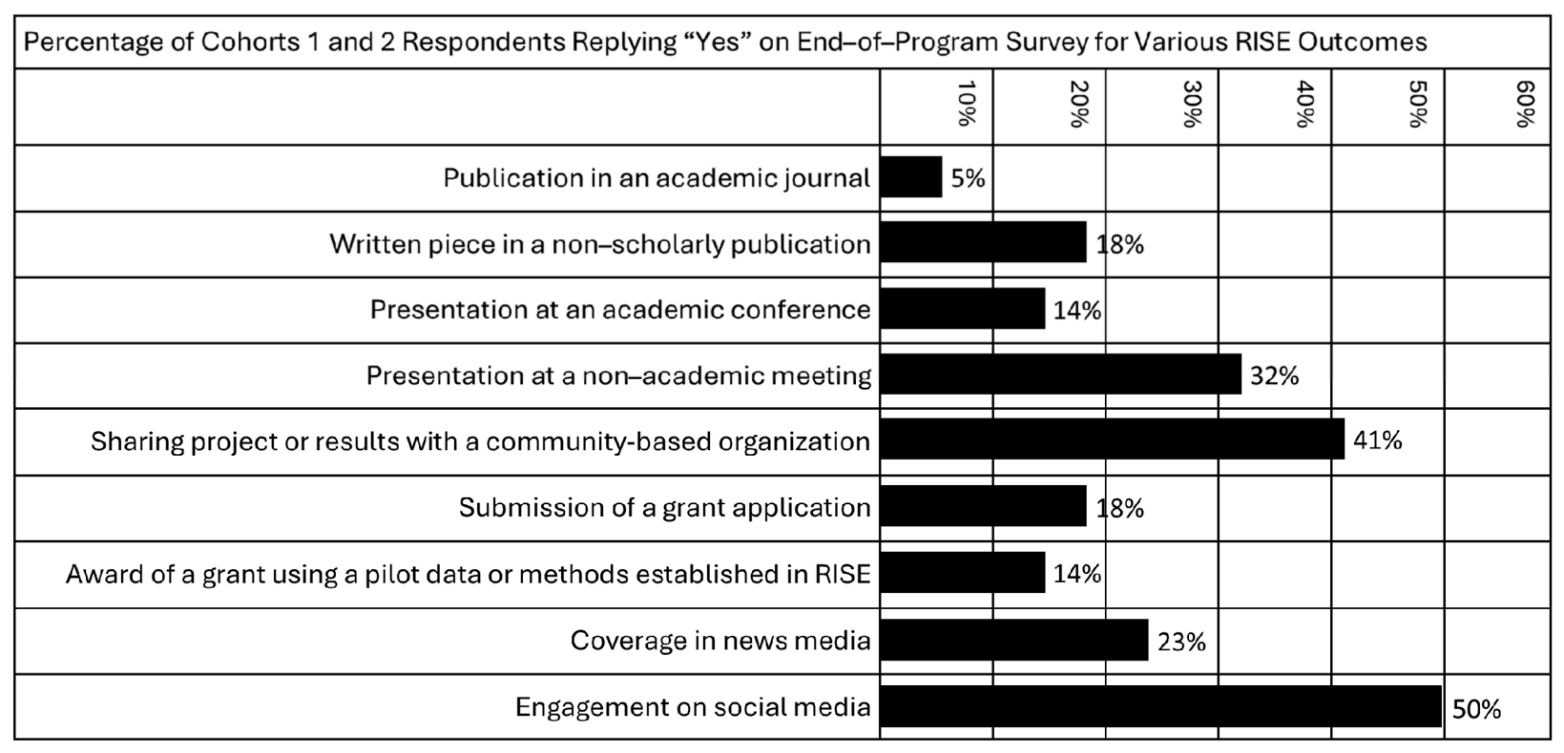
Personal or organizational outcomes reported by RISE Communities participants in the one-year follow-up survey.

Participants also reported successful personal, legislative, or research outcomes associated with their projects (Figure 4). Fifty percent of respondents reported engagement on social media, 41% reported sharing their project or results with a community-based organization, and 32% reported a presentation at a non-academic conference; further explanations of these outcomes on the REDCap surveys showed that many cohort members presented informational talks at community meetings or to stakeholders.

Participants noted that other forms of information sharing not previously stated above included educational dashboards, programs in high school science classes, and providing a training module for students on a Tribal reservation. Participants were also asked to discuss whether they would recommend the RISE training program to others and why or why not. In total, 100% of participants said they would recommend the program. Participants consistently commented on the thoroughness and well-roundedness of the training. Participants also reported that it was helpful to meet other teams and professionals and to be able to learn from each other. Several participants stated that this would be a helpful place to start for organizations curious about starting their own air quality monitoring programs.

The response rate for participants responding to webinar evaluation surveys was 25%. The monthly webinar evaluations revealed that the participants assigned the highest ratings to the “expertise of the presenter(s)” category and assigned the lowest ratings to the “active involvement of participants in the learning experience” category. Overall, the average ratings for each evaluation category of the webinar series increased from cohort 1 to cohort 2.

### 3.3. Focus Groups

Seven members of cohort 1 (four academic partners and three community partners) and three members of cohort 2 (one academic partner and two community partners) participated in the end-of-program focus groups. Qualitative data obtained from the focus groups demonstrated several key takeaways regarding the structure and content of the RISE Communities training program. Focus group participants across both cohorts identified several strengths of the RISE Communities program, including the in-person programming, easy access to experts, increased comfort with community-based research, and enhanced team and community relationships. All participants agreed the provision of air sensors was a critical facilitator in their ability to undertake this type of research. However, trainees also expressed a continued need for training in the technical aspects of air monitoring (from initial sensor set-up to continued deployment over time), sensor data collection and management, and help with managing project timelines, particularly when research team members face competing demands for their time. Participants favored in-person activities and cited the ability to form stronger relationships with their team members, visit community partner sites, and have live demonstrations of air quality monitors. Participants stated that the program allowed for team building. The community-based research components of the training enhanced their ability to connect with the communities they served. When asked to define success and whether their projects had been successful, participants offered a range of responses from deploying air sensors in their communities, training community members in air monitoring, and acquiring funding to continue their efforts. Most participants reported that their goals had shifted throughout the year, but they felt their projects had been successful overall. Participants also reported continuation of RISE projects and project growth beyond initial proposals. Recommendations for program improvement varied by cohort, with the first cohort suggesting that the data training be more tailored to the audience, potentially via breakout sessions for academic partners versus community partners, and more time for teams to work together on their projects. In cohort two, participants requested a more in-depth introduction to different types of air quality monitors with more discussion of the pros and cons of each and enhancement of the engagement and interactivity in the virtual webinars.

## 4. Discussion

### 4.1. Discussion of Findings

RISE Communities is a unique training program intended to promote community–academic partnerships focused on air quality in affected neighborhoods. Its content and training methods are expected to be generalizable to individuals that are concerned about air quality in their home community. Participants reported statistically significant increases in confidence regarding air quality fundamentals, low-cost air sensor usage, and community-engaged research, as evidenced by pre-, post-, and one-year follow-up survey data. In addition, participants reported significantly increased confidence levels in using PurpleAir monitors in their communities, accessing the data from these monitors, and visualizing the data in the short-term follow-up. This is an especially important outcome because technical challenges related to sensor set-up, use, and data collection may be a roadblock for community and academic organizations looking to deploy low-cost sensors to engage their communities in air quality research [24]. Long-term retention of confidence in using these low-cost air quality monitors was lower, correlating to the complex technicality and need for persistent practice using these tools [25].

Community-engaged research is an essential approach to ensure community voice and lived experiences are recognized in research and to increase the impact of research to improve individual and community health [11,16,26]. Studies investigating the impact of this community work on research topics such as air quality have shown promising outcomes for engaging young people in advocacy, increasing the skill bases of communities, and providing more insight into how these issues impact the daily lives of the people affected by them [10,15,27]. The findings in this report further evaluate the long-term efficacy of training community members in the technical and teamwork skills needed for a community-based research project. This study also provides a comprehensive review of the short- and long-term experiences that a training program such as RISE Communities can provide to academic–community research partners; something that is not well documented in the literature.

Another community air quality monitoring project out of Denver noted technical and language barriers with their monitors, citing a lack of user-friendly interfaces and need for more technical training [28]. This is a barrier similarly faced and discussed in our study findings. Community members also seem to have trouble extrapolating the raw data from monitoring into conceptual ideas about pollution, particulate matter, or how environmental actions impact these numbers [29]. Furthermore, participants relate air quality findings to their own personal actions or environments more often than they apply them broadly to an entire community, indicating disconnect between the principles of community science on air quality and how participants actually use their monitors [28,29]. Another study continued to explore this idea, finding that participants who hosted monitors wanted to know how the data would personally impact them, reinforcing the importance of clear expectations around the limits and viability of the data collected from community monitoring [29]. Future air quality monitoring programs should provide clear expectations about how data can realistically be used, explain the collaborative role of a citizen scientist, and provide reminders about the inherent advocacy work that frequently underpins community air monitoring programs.

Results of this study indicate that intensive training in community-engaged research, team function, and air sensor data collection and analysis can contribute meaningfully to both short- and long-term gains in confidence using such methods. The community partners in this study demonstrated less retention of confidence in data analysis, data visualization, and research partnership skills when compared to academic partners. These results suggest that community members’ learning needs may be different from their academic partners, and that a training program in air sensor technology may need to provide more tailored learning opportunities for both audiences. Despite this, both academic and community member participants in this study provided overwhelmingly positive feedback on the program, citing its availability of experts for questions and brainstorming, in-person introduction to fellow team members and organizations, and continued guidance and check-ins during the year as critical components that contributed to the success of their projects.

### 4.2. Limitations

In addition to its strengths, there are some limitations to this analysis that should be considered. First, our sample included 31 participants who were members of the first two cohorts of a five-year training grant. Additional data will be collected in cohorts 3–5 and evaluated separately. Webinar evaluation surveys had low response rates overall, limiting our ability to draw meaningful conclusions about the value and quality of the webinar training, from the participant perspective. Participants in cohort 1 indicated survey fatigue, so we reduced the number of surveys in cohort 2, but this fatigue could have contributed to lower response rates for the monthly webinars. The pre-, post-, and one-year follow-up surveys were developed for pragmatic evaluation of the program. Questions were based on program goals and learning objectives, but were not piloted or tested for reliability, which is another limitation of this study. Due to the nature of the program, we could not reasonably include a comparison group to determine differences in experience from the RISE Communities participants compared to community–academic partners who undertook similar projects without a formal training program to support them. Additionally, the targeted program content potentially introduces a self-selection bias whereas participants enter with specific intent and motivations that may be reflected in evaluation results. Finally, the results of this study are specific to methods and trainees in the RISE Communities program but should be generalizable to individuals that are concerned about air quality in their home community.

## 5. Conclusions

This mixed methods evaluation study found that RISE Communities participants consistently reported increased confidence levels regarding using low-cost air sensors, data manipulation, and understanding community engagement in research immediately following the formal training and at the one-year follow-up. Our study also demonstrated that the partnerships formed during the training resulted in tangible outcomes such as presentations, publications, and grant applications. Constructive feedback centered around the challenges of data management and visualization, demonstrating a further need for instruction on the technical aspects of low-cost air sensor research projects. Future training programs should consider this program’s participant feedback on the difficulty of data management and devote more training time to this topic, revisit the topic often, and offer separate or individualized training to participants such as community members who may not have any background in the technical aspects of research. Because we did not see significant retention of data skills when observing only community partners, future studies may investigate how a tailored training impacts retention of these data skills. This study offers a promising look into how a formal training program in community-engaged air quality research using low-cost sensors can positively impact community–academic research teams.

## Figures and Tables

**Table 1:** Questions assessing confidence in pre-, post-, and one-year follow-up surveys. These are the eight questions that made up the bulk of the statistical analysis in this study. They appeared in the three main surveys evaluated in this study.

Please indicate your level of confidence with the following areas of knowledge or skill: Response options were: (1) Not at all confident, (2) A little confident, (3) Pretty confident, (4) Extremely confident
Describing the health risks associated with air pollution.
Using PurpleAir sensors to collect air quality data.
Effectively participating in a research partnership between a community organization and an academic institution.
Understanding the challenges faced by ‘environmental justice’ communities.
Accessing data received from PurpleAir sensors placed in a community.
Analyzing data received from PurpleAir sensors placed in a community.
Summarizing air quality data in a visual way for others.
Engaging community members in environmental health research.

**Table 2:** Additional questions asked in the one-year follow-up survey. These are questions that only appeared in the one-year survey to gain data on the additional goals teams had reached throughout the year.

Please select any items that have resulted from your RISE Communities project:	1, Publication in an academic journal 2, Written piece in a non-scholarly publication (e.g., newspaper article, blog) 3, Presentation at an academic conference 4, Presentation at a non-academic conference (e.g., Midwestern Sustainability Summit) 5, Sharing project or results with a community-based organization (e.g., giving a presentation or distributing written documents to community members) 6, Submission of a grant application using pilot data or methods established in RISE project 7, Award of a grant using pilot data or methods established in RISE project 8, Coverage in news media (e.g., video news story) 9, Engagement on social media (e.g., tweets, shares, comments) 10, Engagement with policy makers, legislators, or legislative bodies (e.g., the EPA)
If your RISE project resulted in any other forms of information sharing that are not listed in the question above, please describe them here:	
Does your team intend to continue to work on the project that you started with RISE?	1, Yes 0, No 2, I’m not sure
Does your team intend to start a new project using the skills you have learned through the RISE program?	1, Yes 0, No 2, I’m not sure
Would you recommend the RISE Communities training program to others?	1, Yes 0, No
Why or why not?	

**Table 3:** Demographic composition of cohorts 1 and 2. The total number of trainees within each demographic category is labeled under the ‘No.’ column. This number is also represented as a percentage of the entire cohort under the ‘%’ column.

	No.	%
**Gender Identity**		
Woman	23	74%
Man	7	23%
Non-binary, transgender, or gender non-conforming	1	3%
**Hispanic or Latino**		
No	26	84%
Yes	4	13%
Prefer not to report	1	3%
**Racial Background**		
White	16	52%
Black or African American	7	23%
Prefer not to report	6	19%
American Indian or Alaska Native	1	3%
Asian	1	3%
**English As First Language**		
Yes	27	87%
No	4	13%
**Has A Disability**		
No	27	88%
Yes	2	6%
Prefer not to report	2	6%
**Disadvantaged Background**		
No	15	49%
Yes	14	45%
Prefer not to report	2	6%
**Highest Academic Degree**		
Master’s degree	11	35%
Doctorate degree	10	33%
Bachelor’s degree	8	26%
High school/GED	2	6%
**Type of Partner**		
Community	18	58%
Academic	10	33%
Local government official	2	6%
Student at a university	1	3%

**Table 4:** Significance ratings (*p* < 0.05) and effect sizes when cohorts are separated into community partners and academic partners. Pairings labeled (NS) indicate pairings that were not found to have statistically significant changes in confidence levels. Effect size is labeled as (d).

Confidence Statements	Academic Pre- Vs. Post- Confidence Change (CI)	Community Pre- Vs. Post- Confidence Change (CI)	Academic Pre- Vs. One-Year Confidence Change (CI)	Community Pre- Vs. One-Year Confidence Change (CI)
Describing the health risks associated with air pollution	NS	NS	*p* = 0.011, *d* = 1.21	NS
Using PurpleAir sensors to collect air quality data	*p* = 0.03, *d* = 1.23	*p* = 0.013, *d* = 0.68	*p* = 0.001, *d* = 1.85	*p* = 0.002, *d* = 1.06
Effectively participating in a research partnership between a community organization and an academic institution	NS	NS	NS	NS
Understanding the challenges faced by ‘environmental justice’ communities	NS	NS	*p* = 0.007, *d* = 1.32	NS
Accessing data received from PurpleAir sensors placed in a community	NS	*p* = 0.013, *d* = 0.68	*p* = 0.001, *d* = 1.87	*p* = 0.002, *d* = 1.0
Analyzing data received from PurpleAir sensors placed in a community	NS	NS	*p* = 0.003, *d* = 1.62	NS
Summarizing air quality data in a visual way for others	NS	NS	NS	NS
Engaging community members in environmental health research	NS	NS	NS	NS

## Data Availability

The raw data supporting the conclusions of this article will be made available by the authors on request.

## Supplementary Materials

The following supporting information can be downloaded at: https://www.mdpi.com/article/10.3390/ijerph23081051/s1.

## Abbreviations

The following abbreviations are used in this manuscript:

RISE	Research Innovations using Sensor Technology to Improve Community Health
PM_2.5_	Particulate matter that is equal to or smaller than 2.5 micrometers
NAAQS	National Ambient Air Quality Standards
CI	Confidence interval

## Appendix A

### Focus Group Guide

Hi everyone, thank you so much for joining, and welcome to the focus group. Let me introduce myself. My name is ________________ and I’m a Medical Student. I joined the RISE Communities project this summer and have been asked to help gather feedback on your experience with the RISE Program. I’m just going to ask you all some questions on how your experience went and how we can make it better for future participants. I do want you to know that we’re recording this, but no one will see the recording except for me and one other evaluator who is also a part of the program. We will primarily use the written transcript to review your comments and make recommendations to the program directors, and the transcript won’t have any identifying information about who said what. Hopefully you feel free to be open and honest since we will only report overall themes from our discussion, not individual responses. I ask that you also not discuss what others say outside of this room. That being said, if you do not want to answer a question, you are not required to do so.

RISE Project

1. So, overall how would you characterize your experience with the RISE program?
2. RISE is essentially a year-long program. So if you think back to when you started last summer, looking ahead to now, a year later, how would you have defined successful completion of the program for your team at that time?

(a) With your definition of success in mind, would you say that your team was successful in carrying out your project? Why or why not?

3. Please describe any strengths of your project and how RISE contributed to those strengths?
4. Now think about what didn’t work so well in your project. If you encountered any barriers, what did you do to adjust to these?

(a) How did the RISE program help support your team in overcoming those barriers? Or if the program didn’t help, how could have it helped?

5. Tell me about the future of the project you began in RISE Communities. Will what you have started continue?

(a) If not, why not?

(b) If so, how will it continue? What are your objectives for the next 1–3 years with regard to continuing your RISE project?

6. Do you or does your team intend to use the skills you have learned through the RISE program for work outside of your RISE project? For example, data collection, data visualization, analysis, co-locating air monitors, community-engaged research, setting up air monitors. If yes, how do you plan to use these skills?

RISE Program

1. What aspect of the RISE program do you feel was most useful to your learning? (in-person training last summer, monthly webinars, website materials, emails or meetings with RISE faculty or other trainees outside of regular program activities, or something else)
2. Were there any topics or methods of training that you felt were missing from the program?
3. What suggestions for improvement do you have that would help the program directors to improve training for future cohorts?
4. Would you recommend the RISE Communities training program to others? Why or Why not?

Team

1. Were there any changes in the composition of your team during your time with RISE? If so, how did that impact your project?
2. How did the RISE program impact the strength/cohesiveness of your team?

(a). How were the training sessions useful (or not useful) in team development? These sessions could have been Team Development during the in-person training, or webinars such as Creating and Maintaining Strong Community-Academic Partnerships or Aligning community and academic goals.

3. Will the partnerships that you’ve created during the program continue after your conclusion w/RISE?
  a. If not, please describe why.
  b. If so, what are your future plans together?
4. Do you have any additional comments?
